# Exploring the impact of pharmacist-supported medication reviews in dementia care: experiences of general practitioners and nurses

**DOI:** 10.1186/s12877-024-05124-9

**Published:** 2024-06-14

**Authors:** Catharina Carlqvist, Mirjam Ekstedt, Elin C. Lehnbom

**Affiliations:** 1 Department of Health and Caring Sciences, Faculty of Health and Life Sciences, Linnaeus University, Kalmar, Sweden; 2 Department of Pharmacy, Faculty of Health Sciences, UiT The Arctic University of Norway, Tromsø, Norway

**Keywords:** Clinical pharmacy, Collaboration, Dementia, Drug-related problems, Health care professionals, Independent living, Implementation, Medication review, Polypharmacy, Qualitative study, Special housing

## Abstract

**Background:**

Dementia is a major global public health challenge, and with the growing elderly population, its prevalence is expected to increase in the coming years. In Sweden, municipalities are responsible for providing special housing for the elderly (SÄBO), which offers services and care for older individuals needing specific support. SÄBO is both the person´s home and a care environment and workplace. Polypharmacy in patients with dementia is common and increases the risk of medication interactions. Involving clinical pharmacists in medication reviews has been shown to enhance medication safety and improve prescribing practices. However, the views of the standard care team involved in medication prescribing, administration, monitoring and documentation on integrating pharmacist services have received less attention. Thus, this study aims to explore how pharmacists’ contributions can enhance medication safety, improve patient care efficiency, and potentially alleviate the workload of general practitioners for people with dementia living in special housing.

**Methods:**

This study has a descriptive qualitative study design using semi-structured interviews and qualitative content analysis. The study was conducted in a southern Swedish special housing and included nurses, assistant nurses, general practitioners (GPs), and a pharmacist. Due to the COVID-19 pandemic, interviews were conducted over the phone. The Swedish Ethical Review Authority approved the study.

**Results:**

The analysis revealed three main categories, and eleven subcategories.: (1) Integrating multidisciplinary approaches for holistic dementia care, (2) Strengthening dementia care through effective medication management and (3) Advancing dementia care through pharmacist integration and role expansion. Nurses focused on non-pharmacological treatments, while GPs emphasized the importance of medication reviews in assessing the benefits and side-effects of prescribed medication. Pharmacists were valued for their reliable medication expertise, appreciated by GPs for saving time and providing recommendations prior to consultations with individuals with dementia and their next-of-kin. Although medication reviews were considered beneficial, there was skepticism about their ability to solve all medication-related problems associated with dementia care.

**Conclusions:**

This study highlights the critical role pharmacists play in enhancing medication safety and patient care efficiency in special housing for individuals with dementia. Despite the value of their contributions, communication barriers within healthcare teams pose significant challenges. Recognising potential pharmacist role expansion is essential to alleviate the workload of GPs and ensure effective collaborative practices for better patient outcomes.

**Supplementary Information:**

The online version contains supplementary material available at 10.1186/s12877-024-05124-9.

## Background

Dementia is a term used to describe several chronic and progressive diseases that cause significant damage to the brain. Depending on which parts of the brain are affected, dementia can manifest itself in different ways, but common symptoms include impaired memory, language deficits, problems with time perception and loss of orientation skills [1]. Dementia can also cause changes in behavior, such as increased anxiety and aggression, which can be challenging for family and caregivers to handle [2]. As a result, special housing for the elderly (SÄBO) has been established in Sweden to provide apartment-based care that aims to create a supportive environment for individuals with dementia. SÄBO is both the person´s home and a care environment and workplace [3].

According to the World Health Organization (WHO), over 55 million people worldwide are currently living with dementia [4], with 130,000–150,000 cases in Sweden alone. As the elderly population continues to grow, the prevalence of dementia is expected to increase in the coming years [5]. In Sweden, local municipalities are responsible for providing care to residents in special housing, as outlined in the national Social Services Act. Healthcare professionals who provide direct patient care in special housing typically include enrolled nurses, nursing assistants with upper secondary level schooling, and registered nurses. Registered nurses often delegate the administration of medications to enrolled nurses and nursing assistants who have training in medication administration [6].

There are several medications available to treat the symptoms of dementia. Besides dementia medication that may slow cognitive decline, there is frequent use of antipsychotics, benzodiazepines, and sleep medications, which can be potentially inappropriate for these individuals due to increased risks of side effects and further cognitive impairment [7]. Since many people with dementia also have other chronic conditions [8], they are at a high risk of polypharmacy. Polypharmacy refers to the concurrent use of multiple medications, which can increase the likelihood of potentially dangerous drug interactions or undesirable side effects. While there is no exact definition of the number of medications that constitutes polypharmacy, it typically refers to the use of five or more medications [9].

It is crucial to take steps to optimize medical treatment for individuals with dementia [10]. This can be achieved by carefully selecting pharmacological and non-pharmacological treatments, stopping medications that are no longer needed, and adjusting dosages to minimize or prevent drug-related problems. Medication reviews, which involve a structured evaluation of a patient’s medications, have been recommended as a method to identify and resolve drug-related harm [11, 12]. According to regulations from the Swedish National Board of Health and Welfare [13], patients aged 75 years or older who are prescribed five or more medications have the right to receive a yearly medication review with a physician. This helps ensure that their medications are being used safely and effectively.

Previous studies have demonstrated that involving clinical pharmacists in medication reviews can result in safer medication use in various populations [14], including individuals with dementia [15]. However, healthcare professionals’ perspectives on the implementation of medication reviews and other pharmacist services have received less attention. This is crucial because successful implementation depends on their acceptance, given factors such as time constraint and information [16]. In a previous qualitative study, general practitioners (GPs) in Sweden expressed a positive attitude towards collaborating with clinical pharmacists in hospitals [17]. However, they also emphasized their autonomy as decision-makers and the need for more information about the service, such as how to get in touch with the pharmacist when needed.

The management of a Swedish county recently proposed introducing clinical pharmacists to conduct medication reviews for people with dementia living in special housing to optimize impact of medication treatment. However, a top-down approach may result in less employee commitment to new ways of working compared to a bottom-up approach, where employees are involved and have input in the process [18]. Therefore, it is critical to understand the perspectives and experiences of healthcare professionals when evaluating the success of the new work process.

In this study, representatives of healthcare professionals closely involved in a patient’s care and medication management at the special housing were interviewed to gain a comprehensive understanding of their experiences in working with a pharmacist. The aim was to explore healthcare professionals’ experiences of pharmacist-supported medication reviews for people with dementia living in special housing.

## Methods

### Study design

The study used a descriptive qualitative design, involving semi-structured interviews that were analyzed using qualitative content analysis guided by Graneheim and Lundman [19].

### Setting

The study was conducted in special housing for dementia care located in a municipality in southern Sweden. In Sweden, it is common for people with dementia, who can no longer manage in their private homes, to live in special housing administered by municipalities. These facilities are under municipal administration, while GPs, who are employed at healthcare centers, are under county council administration. Since 1996, the non-profit organisation Silviahemmet Foundation has offered a one-year course in advanced dementia care at Sophiahemmet University nationally. After completion, registered nurses and assistant nurses receive the exclusive title Silvia Nurse or Silvia Sister, conferred by HM Queen Silvia [20]. In this study, Silvia Nurses and Silvia Sisters worked in the special housing located in the southeast region of Sweden, and all interviewed GPs were medically responsible for one or more patients in these facilities The pharmacist, employed by the county council, provided medication review services to patients in special housing as part of the management’s approach to optimize treatment impact for people with dementia.

### Sampling and recruitment of participants

A purposive sampling strategy was used to identify and recruit healthcare professionals who provide care in special housing for individuals with dementia. Eligible healthcare professionals included registered nurses, assistant nurses, GPs, and pharmacists. In addition to working at the special housing or being medically responsible for a resident, criteria for participation also required individuals to have been involved in at least one medication review.

This study was conducted within the context of a relatively new initiative focusing on medication reviews in dementia care. The leadership informed healthcare professionals about the study, and contact details were forwarded to the researchers. ECL provided further information about the study to potential informants, and all but one GP agreed to participate in the study. The interviewed GPs were either specialists in general medicine or were in training to become specialists. All registered nurses and assistant nurses had qualifications in caring for individuals with cognitive decline and all held a Queen Silvia (Assistant) Nurse Diploma.

### Data collection

A semi-structured interview guide was developed by ECL and ME to explore how healthcare professionals involved in multi-disciplinary medication reviews viewed the process and potential outcomes. The themes covered in the interviews included:

Professional perspectives on medication management – to explore the views of healthcare professionals on the balance between medication treatment and care, addressing discrepancies in medication management, and the challenges and benefits of continuity in patient care.

Collaborative practices in dementia care – to understand the dynamics of family interactions, collaboration between healthcare professionals, and the role of pharmacists in optimizing medication safety.

Interdisciplinary teamwork and role expansion – to investigate the integration of pharmacists into multidisciplinary teams, the potential for expanding pharmacist roles to support GPs, and the overall impact of pharmacist-supported medication reviews on enhancing patient care.

Due to the COVID-19 pandemic and the location constraints, as the researcher (ECL), a female pharmacist with a PhD, was based in a different city than the informants, the interviews were conducted over the phone. ECL, who has extensive training in qualitative methods, conducted, took notes and audio-recorded all interviews. Verbatim transcription was done by a professional and checked for accuracy by ECL. A total of four GPs, two nurses, two assistant nurses and one pharmacist were interviewed. The interviews’ duration ranged from 18 to 46 min, with an average length of 32 min. Participants were not reimbursed for their time as interviews took place during office hours.

### Data analysis

The interview data were analyzed using qualitative content analysis guided by Graneheim and Lundman [19], led by CC. First, the verbatim transcripts were read thoroughly several times to gain an overall understanding of the data. Meaningful units containing sentences or phrases related to the aim were identified and labeled with descriptive codes close to the text.

Next, the codes were compared and contrasted with other codes, and similar codes were merged. Related codes were classified into subcategories, mainly reflecting the manifest content of the text. Finally, broader main categories were formed by grouping related subcategories together. The analysis was iterative, with codes, subcategories, and categories continuously modified during the analysis process.

To increase trustworthiness of the results, there was an ongoing dialogue among all the authors throughout the analysis process. Refinements were made until agreement was reached about the content of the analysis.

### Ethics

Ethical approval was granted by the Swedish Ethical Review Authority (2019–06322), and the Consolidated Criteria for Reporting Qualitative Studies (COREQ) [21] was used to report the findings of this study.

## Results

The results describe healthcare professionals’ (GPs, pharmacist, nurses and nurse assistants) experiences of medication reviews conducted by a clinical pharmacist for individuals with dementia. The results are organized into three categories and 11 subcategories identified in the analysis, as shown in Table 1.

**Table 1:** Categories and subcategories extracted from content analysis

Categories	Subcategories
Integrating multidisciplinary approaches for holistic dementia care	Valuing interprofessional collaboration and education in dementia care Communication barriers in collaborative dementia care Challenges and benefits of continuity in dementia patient care Family dynamic and communication in dementia care
Strengthening dementia care through effective medication management	Balancing medication care in dementia treatment Addressing discrepancies in medication management The role and challenges of medication reviews in dementia care
Advancing dementia care through pharmacist integration and role expansion	The impact of pharmacist involvement in medication management Integrating pharmacists in comprehensive care Expanding pharmacist roles to enhance patient care and support GPs

### Integrating multidisciplinary approaches for holistic dementia care

#### Valuing interprofessional collaboration and education in dementia care

The work of the pharmacist was mostly appreciated by the standard healthcare team, and the pharmacist experienced that GPs were grateful for the work done. It was believed that pharmacists have an important role in meeting primary care needs, and the introduction of municipality pharmacists was believed to achieve better drug use.

One nurse described that the project had been educational and had led to greater vigilance regarding medication treatment. The discussion climate was mostly described by participants as open and respectful, and building personal relationships through physical visits was identified as an important facilitator.

*It has been very positive, partly for my own part, that you have learned a lot and started to reflect a lot on the medication that the person uses, that you should always think about whether they are current, and so on*. IP9, nurse

One GP hesitated to implement the pharmacist’s recommendation without further consideration, as they were responsible for the treatment. Not all GPs shared this view, and some appreciated the input from the pharmacist and the ability to discuss pharmacotherapy with another healthcare professional.

When asked for possible solutions or other ways of collaborating, the pharmacist suggested more resources in terms of personnel so that different healthcare professionals could meet, discuss, and agree on a solution, rather than communicating through messages. This would facilitate more effective collaboration and reduce the need for double work. However, one nurse felt that they were less listened to by physicians, indicating a need for further improvement in communication and collaboration between healthcare professionals.

### Communication barriers in collaborative dementia care

During the interviews, participants highlighted how communication as a transfer of information affected collaboration and the ability to see the overall picture. Both the pharmacist and a nurse raised concerns about communication difficulties with GP. A barrier discussed was the lack of direct communication between the pharmacist and GPs, which obstructed effective collaboration.

After conducting a medication review, the pharmacist entered medical notes into the electronic medical record and sent a digital message to the GP. However, a shortcoming described was the lack of possibility to nuance recommendations. Nevertheless, the pharmacist expressed a willingness to be more involved and easily accessible for GPs for further communication.

Deficient communication between GPs and nurses was also described. Changes in medication could also occur without notice, posing a risk to patient safety, as reported by the nurse. Internally there were questions about the GPs willingness to initiate contact with colleagues. Additionally, GPs reported not always receiving feedback after specialist care at referral hospitals, which made it difficult to maintain control over patients’ current medication regimen. Despite established digital communication methods, obtaining sufficient information from hospitals about patients’ medication and treatment remained a challenge for GPs.

*It has happened, on several occasions, that the doctor has made medication changes without my knowledge, and I later find out that they have either discontinued or added a medication. … This is a risk*. IP9, nurse

### Challenges and benefits of continuity in dementia patient care

Most of the healthcare professionals in the study emphasized the importance of continuity of care. They raised concerns about patients seeing multiple GPs’, which could interfere with the treatment plan and compromise patient safety. Seeing the same patients over time was described as making the work more efficient for GPs and contributing to patient safety.

However, one nurse pointed out that patients’ right to choose a GP constitutes an obstacle to having one GP, preferably someone with specialist training and interest in dementia care, attend to all individuals with dementia in special housing. Instead, nurses had to liaise with many different GPs, some of whom had a strong interest in geriatric patients and patients with dementia while other GPs were more interested in other patient populations.

While the clinical pharmacist performed medication reviews, the GPs’ visited the residents in special housing, promoting continuity of care and improving medication safety. This continuity of care also extended beyond GPs, as all medication reviews in this project were conducted by the same clinical pharmacist. Nurse assistants and registered nurses appreciated this, as it allowed them to become familiar with the pharmacist, understand their role and what they could contribute.

*Continuity is also important; perhaps ensuring that it is the same pharmacist who has an understanding and an agreement. This way, there won’t be different pharmacists involved, which can make it a bit challenging for the doctors to maybe maintain this continuity with the pharmacist. It’s crucial, I would say, to ideally have the same pharmacist who handles the same residences, the same patient groups, and the same doctors. This makes it more efficient and safer for all of us.* IP2, GP

### Family dynamic and communication in dementia care

The relationships that staff were able to form with the residents and their next-of-kin were identified as other factors that influenced patient care. Caring for individuals with dementia can be complicated because they are often unable to communicate what is troubling them.

*And then it’s very difficult to discontinue these medications. Either the patient protests or the relatives protest, and it’s very challenging.*. IP1, GP

One GP reported that it was sometimes difficult to discontinue medications due to reluctance from either the individual with dementia or their next-of-kin. On the other hand, one nurse reported that most relatives felt comfortable handing over the responsibility of medication management to healthcare professionals. This was because the relatives were often exhausted from caring for someone with dementia over a long period of time.

### Strengthening dementia care through effective medication management

#### Balancing medication and care in dementia treatment

Nurses emphasized non-pharmacological treatments when discussing treatment options. Their experiences indicated that pharmacological treatment was not always the optimal choice for individuals with dementia. Pain was as an example where nurses instead favored a greater focus on the caring aspects as a more suitable and safer approach to relieve symptoms.

*We strive for individuals with dementia to have as few medications as possible*. IP6, nurse

Nurses also expressed concern about the overuse of antipsychotic medications, which they felt were sometimes prescribed for the wrong indication. They also raised concerns that not all individuals with dementia were receiving anti-dementia medications, which they believed more individuals would benefit from.

Nurses attributed some GPs’ lack of specialist training in dementia care as a major barrier to making appropriate medication decisions. However, they also acknowledged that some GPs commitment and knowledge were enablers for achieving safe and appropriate medication use.

*I actually think, unfortunately, that the knowledge among our general practitioners here at the healthcare centers is not really there either, with dementia medications and such, and then they resort to these sedative medications instead.*. IP6, nurse

Nurse assistants perceived that they had detailed knowledge about the individuals, which was vital for GPs to make informed decisions about medication regimens. However, they felt frustrated that their input was not fully utilized. They rarely met with GPs and instead provided their input to the nurse, who may or may not have brought it up with the GP. Nurse assistants were concerned that misinterpretation of an individual’s symptoms could lead to inappropriate medication interventions, but this concern was not reflected in the GPs’ responses.

GPs, on the other hand, seemed more focused on continuous monitoring after starting a new medication or making a dose change. They emphasized the importance of evaluating a patient’s medication regimen to prevent or avoid possible harms of medications.

*But I think it’s important in the long run with follow-up, that every time I start a new blood pressure medication, there is a follow-up after 30 days, whether you like it or not, whether it’s over the phone or in person.*. IP8, GP

### Addressing discrepancies in medication management

Several healthcare professionals in the study expressed concerns about incomplete and inaccurate medication and prescription lists. Nurses described how they used medication administration lists to administer medications to residents, which could be different from the list of current prescriptions.

*If a medication is initiated in the hospital, there must be a clear … discontinuation date or plan when discharging the patient, to ensure that medications that are not meant to continue are removed from the medication list and discontinued*. IP2, GP

GPs acknowledged their responsibility to cancel current prescriptions when a medication has been ceased or there is a dose change. This was seen as a way to improve the accuracy of medication lists and prevent medication errors.

Having access to the same information was also considered to be of great importance. Some healthcare professionals mentioned that they shared the same medication lists, which provided a common ground. The pharmacist recognized that the dose-dispensing system provided a low risk for discrepancies.

### The role and challenges of medication reviews in dementia care

Healthcare professionals viewed medication reviews as a meaningful method for improving patient treatment in dementia care. The pharmacist highlighted that medication reviews could provide an opportunity to optimize a patient’s overall treatment plan. One nurse stressed the importance of reviewing all medication when a new individual moves into special housing. While medication reviews were considered useful, one GP expressed skepticism that they could solve all dementia-related problems.

GPs viewed performing medication reviews as a natural component of patient visits for assessing the benefits and side-effects of prescribed medication, particularly for older adults who are prescribed multiple medications. However, GPs differed in their approach to when and how to conduct medication reviews.

In most cases, performing a simple medication review was regarded as sufficient, and a comprehensive medication review was almost never considered necessary. However, sometimes an initial simple medication review would evolve into a comprehensive review when needed. During a medication review, GPs recognized the importance of considering clinical data, side effects, and the risk of interactions. GPs described the procedure as a thorough evaluation of all medications, in discussion with the patient when possible. This was also an opportunity to identify and correct potential medication discrepancies.

*So, I go through each medication, what effects and side effects they may have had on patients. Are there any risks of side effects, such as dizziness and falls, and are there any other side effects that cause suffering for the patient? What is the benefit of this medication? And what is the harm? We weigh the benefits against the harm. Does the patient even need it? How is their kidney function, liver function, and risk of interactions*? IP2, GP

GPs and the pharmacist highlighted the importance of having sufficient time to conduct a meaningful medication review, particularly for complex patients requiring a comprehensive medication review. However, due to time restraints, one GP only considered new medications and not medications the patient had used long-term during medication reviews. A lack of time was perceived as a significant barrier and threat to patient safety. A GP described time constraints like this:

*The biggest threat, which is actually the most important, is that the doctor does not have time to devote to medication review during the meeting with the patient and cannot go through the patient’s treatment*. IP2, GP

One GP viewed involving pharmacists in conducting medication reviews as resource-intensive and resulting in double work, as both the GP and the pharmacist had to document their findings. Additionally, if the pharmacist had identified suboptimal treatment and recommended a change, the GP still had to devote time to understand the problem and consider the recommended solution against other alternatives.

### Advancing dementia care through pharmacist integration and role expansion

#### The impact of pharmacist involvement in medication management

Most GPs viewed pharmacists as reliable healthcare professionals who served as an important source of medication expertise. These GPs appreciated that involving a pharmacist saved time, as they were provided with recommendations and viewpoints in advance of their meetings with patients.

Treatment proposals by pharmacists were often seen as relevant and contributing to patient safety.

*I think that these people [pharmacists]do a great job and make a big contribution to the patient’s medication safety*. IP4, GP

The pharmacist experienced that the GPs’ need for support varied depending on their level of professional confidence. They also pointed out that GPs had not been given the opportunity to discuss with the pharmacist the types of medication related problems they wanted help with. As a result, the collaboration between GPs and the pharmacist was described as suboptimal.

Pharmacists were also found to provide valuable support to nurses and assistant nurses. One nurse believed that it would be valuable for GPs to have the pharmacist as a discussion partner in cases of disagreements, and it was also suggested that GPs would benefit from initiating more contact with the pharmacist. Receiving confirmation from a pharmacist was empowering and made it easier to stand up to GPs in cases of suspected suboptimal treatment.

The pharmacist reported that nurses asked for help with medication administration problems, such as tablet crushing or splitting. Additionally, one assistant nurse stated that it was preferable to ask a pharmacist rather than a GP for medication-related questions. However, the possibility to initiate contact with pharmacists was limited to GPs, and one nurse desired the opportunity to ask the pharmacist directly in case of questionable medication decisions. The pharmacist also received requests to help with continuing education, emphasizing a need for more education and the potential to use pharmacists as a valuable resource of medication knowledge.

*And so, there might be some… some desire for professional development. At least that’s what I’ve been told.*. IP3, pharmacist

This category focuses on the evolution and enhancement of dementia care by incorporating pharmacists into the care team. It considers the transformative potential of pharmacists’ expertise in medication management if fully integrated into multidisciplinary teams and explores the possibilities that arise from expanding their roles within the care continuum.

### Integrating pharmacists in comprehensive care

The pharmacist highlighted how the different professions completed each other by applying pharmaceutical consideration, caring, and medical treatment. One GP pointed out that pharmacists can offer help in discovering medication interactions in patients who have recently been hospitalized.

However, as pointed out by one nurse, the pharmacist needs to take into account the whole story behind the medication choices made and not only have pharmaceutical considerations in mind. Therefore, collaboration with assistant nurses working close with the individual with dementia was described as necessary to aid the pharmacist in making optimal treatment suggestions.

One nurse said that the pharmacist’s questions stimulated reflection on the individual’s medication, and the knowledge learned from the pharmacist was applied to other individuals. Involving a pharmacist at the time of diagnosis was suggested by a nurse to prolong the time at home for patients with dementia and improve their well-being. Furthermore, one GP stated that the use of pharmacist services was most valuable for optimizing treatment in elderly patients (75–85 years of age) with many medications. Consequently, the management of patients with multimorbidity and dementia was said to especially benefit from the support of a pharmacist. One shortcoming that was brought up by both the pharmacist and a nurse was the absence of follow-up on the pharmacist’s recommendations:

*Currently, there is no direct follow-up on those recommendations, uh… regarding whether they benefit the patient in the long run. It would have been very interesting to follow up on how… both the municipal staff and then… relatives and doctors, to see if there has been any difference in the recommendations like that.*. IP3, pharmacist

### Expanding pharmacist roles to enhance patient care and support GPs

One GP mentioned potential areas for future pharmacist work tasks, such as routine prescription renewals and blood pressure measurements, which could reduce workload for the GP. It was also suggested that pharmacists could make home visits to check how patients handle their medications.

*Why couldn’t the pharmacist at least renew the prescription if there is … a good reason and no vital parameters that need to be taken? But again, there could be a communication issue.*. IP8, GP

Furthermore, one nurse believed that it would be valuable for individuals in special housing to have medication reviews conducted by pharmacists, as these patients may receive medical care from many different providers.

## Discussion

The aims to explore how pharmacists’ contributions can enhance medication safety, improve patient care efficiency, and potentially alleviate the workload of general practitioners for people with dementia living in special housing. The main results indicate that healthcare professionals value the expertise of pharmacists, particularly in ensuring medication safety and efficiency in patient care. Communication challenges between pharmacists and other healthcare staff were identified, affecting the optimal integration of pharmacists’ recommendations. There is consensus on the potential benefits of expanding pharmacists’ roles, including routine prescription management and home visits, to better support GPs and enhance patient care. However, a lack of follow-up on the implementation of pharmacists’ suggestions was noted, highlighting a gap in assessing the long-term benefits of involvement in dementia care.

Despite the generally positive views of healthcare professionals towards the contributions of pharmacists and the value of their input in medication management, there was some skepticism regarding the suitability of pharmacist-supported medication reviews for individuals with dementia who live in special housing facilities where they receive care and oversight. This level of monitoring may not require the same level of intervention as needed for other patient groups. Instead, it was suggested that individuals living independently at home, especially those with multiple comorbidities and polypharmacy, might be a more suitable target group for pharmacist involvement. Furthermore, the shortage of GPs, which poses a potential risk to patient safety [22], needs to be addressed. Leveraging pharmacists’ expertise through targeted task shifting could potentially alleviate the pressure on overburdened GPs [23]. While this approach is promising, concerns regarding patient safety have been raised in a Norwegian study examining horizontal task-shifting between specialists with varying levels of expertise [24]. This highlights the importance of careful planning and preparation before implementing task-shifting across professions. To optimize the potential benefits of pharmacist involvement for GPs, it is recommended that GPs proactively identify and refer patients who could benefit most from pharmacists’ expertise, including patients experiencing polypharmacy, side effects, or medication efficacy issues. This targeted approach could ensure pharmacist interventions are directed where they are most needed, enhancing patient safety and care outcomes.

Effective communication within multidisciplinary teams is vital in ensuring high-quality patient care, particularly for vulnerable populations. While the interviewed healthcare professionals recognize the value of pharmacists in medication management, there are communication challenges that hinder the optimal integration of pharmacists’ recommendations. These challenges are not unique to this study; previous research has consistently highlighted the critical role of clear and structured communication channels in multidisciplinary healthcare settings [25, 26]. Opportunities for improvement include establishing regular interdisciplinary meetings and utilizing shared electronic health records to facilitate real-time information exchange. Interprofessional learning, whether at the workplace or as part of students’ education, could be a valuable facilitator for overcoming these barriers [27]. Furthermore, actively engaging patients in communication, where feasible, can personalize care, ensuring medication regimens align with both guidelines and individual needs and preferences, particularly when treatment goals shift from prolonging life to enhancing quality of life [28]. Addressing these communication barriers is essential to fully realize the potential benefits of pharmacist involvement in dementia care, as it would foster a more cohesive approach to medication management and ultimately enhance patient outcomes.

The potential for expanding the role of pharmacists is a topic of growing interest in Sweden, particularly as a strategy to alleviate the increasing workload on GPs and to provide more comprehensive care. This study suggests that pharmacists could assume additional responsibilities, such as managing routine prescription renewals and performing home visits, especially for patients with complex care needs. This proposed expansion mirrors the successful integration of nurse prescribing rights in certain clinical areas [29] and the role of clinical pharmacists in other countries [30, 31]. Empowering pharmacists in a similar capacity would not only enhance the quality of care for patients by ensuring more timely and precise medication management but also contribute to a more efficient use of healthcare resources. By redistribution of certain tasks traditionally performed by GPs to pharmacists, the healthcare system can leverage the specialized knowledge of pharmacists to improve patient outcomes and address the challenges of an overburdened primary care infrastructure.

### Strengths and limitations

Study limitations emerged due to pandemic-related constraints, necessitating a shift from planned face-to-face interviews to telephone interviews. Challenges included maintaining clear communication and interpreting interviewee reactions without visual cues. However, telephone interviews have been shown to facilitate greater participant openness [32, 33] and increased participation, especially among time-constrained or geographically distant groups [34]. Additionally, they allowed more efficient use of economic and human resources, significantly reducing the need for in-person travel.

To enhance dependability, we developed a semi-structured interview protocol that was consistently applied in all interviews. Additionally, all interviews were conducted by the same researcher (ECL), further enhancing consistency in data collection [35]. A diverse range of healthcare professionals who had been involved in medication reviews conducted by the pharmacist were interviewed, offering rich insights into the complexity of medication management among people with dementia. The sample was heterogeneous in terms of professional background, work experiences, and roles in medication management within special housing. Despite the broader diversity, the investigation focus was narrow and concerned their views on medication safety. Importantly, all participants had relevant experience related to the role of pharmacists in medication management, ensuring high specificity for the study [36].

We endeavored to interview all relevant professionals who had participated in medication reviews and found conflicting views, especially among GPs. Thus, all participants had experiences of the procedure, with variation in participant characteristics and experiences to ensure strong data. This may strengthen the transferability of our findings to similar contexts. We believe the data, while derived from a small sample size, are robust enough to provide sufficient information power to elucidate the study’s aim [36].

The trustworthiness and dependability of our findings were further supported by meticulous adherence to accepted research principles during sample selection, data collection, and analysis. To enhance the credibility of the results, the analysis underwent investigator triangulation involving thorough discussions, challenges, and redefinition by all authors, who comes from diverse professional backgrounds (pharmacist and nurse). To strengthen confirmability and credibility further, we support our findings with quotes that provide valuable insights into the participants’ perspective, thus authentically representing their voices [35].

## Conclusion

This study provides valuable insights into optimizing medication management practices, a critical aspect of enabling individuals with dementia to live safely and comfortably at home. While the expertise of pharmacists is highly valued in ensuring medication safety and enhancing patient care efficiency, communication barriers within the multidisciplinary team present significant challenges to fully integrating pharmacists’ recommendations. Recognizing the potential for role expansion among pharmacists, we explore a promising avenue to alleviate the workload of GPs. To fully realize the benefits of pharmacist contributions to dementia care, a thorough evaluation of potential patient safety risks is essential, ensuring both medication safety and efficient patient care. Furthermore, healthcare professionals generally expressed positive attitudes toward collaborating with pharmacists. The study emphasizes the importance of involving healthcare professionals in the implementation of new work processes to secure employee commitment and successful adoption.

### Electronic supplementary material

Below is the link to the electronic supplementary material.

Supplementary Material 1

## Data Availability

The datasets generated and analysed during the current study are not publicly available due to the participants not providing consent for their data to be shared publicly, but are available from the corresponding author on reasonable request.
